# Unraveling the interplay between sleep, redox metabolism, and aging: implications for brain health and longevity

**DOI:** 10.3389/fragi.2025.1605070

**Published:** 2025-05-21

**Authors:** Fayaz A. Mir, Arianna R. S. Lark, Christa J. Nehs

**Affiliations:** ^1^ Mass General Brigham Department of Anesthesiology, Massachusetts General Hospital, Harvard Medical School, Boston, MA, United States; ^2^ Division of Sleep Medicine, Harvard Medical School, Boston, MA, United States

**Keywords:** sleep, mitochondria, oxidative stress, metabolic, antioxidants, ketones

## Abstract

The relationship between sleep and metabolism has emerged as a critical factor in aging and age-related diseases, including Alzheimer’s disease and dementia. Mitochondrial oxidative phosphorylation, essential for neuronal energy production, also generates reactive oxygen species (ROS), which increase with age and contribute to oxidative stress. Sleep plays a vital role in modulating redox balance, facilitating the clearance of free radicals, and supporting mitochondrial function. Disruptions in sleep are closely linked to redox imbalances, and emerging evidence suggests that pharmacological interventions, such as dual orexin receptor antagonists and antioxidant-based therapies, may help restore redox homeostasis. Furthermore, antioxidant-rich diets and supplements have shown promise in improving both sleep quality and metabolic health in aging populations. Neurons, with their high energy demands, are particularly vulnerable to oxidative damage, making redox regulation crucial in maintaining brain integrity. This review explores the bidirectional relationship between sleep and redox metabolism through five key areas: (1) sleep’s role in free radical regulation, (2) ROS as mediators of age-related sleep disturbances, (3) feedback loops between impaired sleep and brain metabolism, (4) sleep, redox, and aging in peripheral systems, and (5) therapeutic strategies to restore redox balance and improve aging outcomes. Understanding these mechanisms may provide new targets for interventions aimed at mitigating age-associated diseases.

## 1 Introduction

Within the field of aging research, the intricate relationship between sleep architecture and metabolism has emerged as a significant contributor to age-related health changes and pathological aging conditions, such as Alzheimer’s disease and dementia (Carroll and Macauley, 2019; Zhang W. et al., 2024). Studies have shown that oxidative phosphorylation of glucose, along with electron transfer chain reactions used to generate energy, also produces substantial free radicals, which are further elevated in the aging brain (Gemma et al., 2007; Butterfield and Halliwell, 2019). Neuronal mitochondrial redox biology and its optimization are essential for overall brain function, and sleep has been shown to regulate and play an important role in scavenging free radicals (Yin et al., 2014; Hill et al., 2018; Richardson and Mailloux, 2023). Imbalances in the brain’s redox potential within the mitochondria can be directly linked to sleep disturbances, and studies have demonstrated that improving sleep with medications like dual orexin receptor antagonists and benzodiazepine-like drugs can help restore redox balance (Singh and Kumar, 2008; Zhang W. et al., 2024). Furthermore, recent clinical and animal studies have shown that oxidative stress can be mitigated by consuming antioxidant-rich foods, supplements, and specialized diets which can, in turn, improve both sleep and overall health (Lei et al., 2023).

As humans, we spend about one-third of our lives sleeping, yet much of the underlying physiology and mechanisms of sleep remain elusive. Sleep plays a critical role in regulating the body’s bioenergetics, particularly in the brain (Richardson and Mailloux, 2023). Studies have highlighted many of sleep’s unique functions: restoring energy levels, synthesizing biomolecules for tissue regeneration, and clearing free radicals, all of which promote an optimized physiological state (Adam and Oswald, 1977; Benington and Heller, 1995; Siegel, 2005; Mackiewicz et al., 2007; Davinelli et al., 2024). Deciphering the bidirectional relationship between sleep and redox bioenergetics and understanding their impacts on aging and age-associated pathologies, is both timely and highly relevant to addressing the current disease burden. These studies will offer vital insights into the complexities of aging and its connection to sleep and metabolism, providing new opportunities for developing better drug targets to reduce age-related health disparities.

Reactive oxygen species (ROS), traditionally viewed as damaging metabolic byproducts, are now recognized as critical signaling molecules in the brain, where they modulate various neural functions including synaptic plasticity, neurogenesis, and circadian rhythm regulation. At physiological levels, ROS play an essential role in redox signaling by influencing the activity of transcription factors such as NF-κB and Nrf2, and modulating pathways like MAPK and PI3K/Akt, which are vital for neuronal survival and adaptation (Finkel and Holbrook, 2000; Sies and Jones, 2020). This redox signaling plays a critical role in maintaining cellular homeostasis and responding to environmental cues. However, when ROS production exceeds the cellular antioxidant capacity, oxidative stress ensues, leading to damage of proteins, lipids, and DNA. In the central nervous system, mitochondria in neurons and astrocytes are key sources of ROS, which help fine-tune neurotransmitter release, long-term potentiation, and cognitive functions like learning and memory (Massaad and Klann, 2011). However, due to the brain’s high oxygen consumption and lipid-rich environment, it is particularly vulnerable to oxidative stress when ROS levels exceed antioxidant defenses. This delicate balance, where low to moderate ROS levels facilitate neuronal signaling and plasticity, but excessive accumulation contributes to neurodegeneration, highlights the brain’s reliance on tightly regulated redox homeostasis (Schieber and Chandel, 2014; Angelova and Abramov, 2016).

Neurons are highly energy-demanding, and their mitochondria are key producers and modulators of oxidative stress, which can have severe consequences if not neutralized by antioxidant mechanisms (Angelova and Abramov, 2018). As we age, mitochondrial function changes, leading to an increase in ROS production over time, along with impairments in antioxidant processes (Cui et al., 2012; Chistiakov et al., 2014; Giorgi et al., 2018; Stefanatos and Sanz, 2018). These changes contribute to ROS-mediated aging and associated sleep-wake disturbances (Davalli et al., 2016). Over time, such changes can lead to several life-threatening diseases, including neurodegeneration and cardiovascular abnormalities (Cappuccio et al., 2011; Feng et al., 2022; Zhang W. et al., 2024). Understanding how redox metabolism is altered in sleep disorders, such as insomnia, neurodegenerative diseases, and aging, may provide crucial insights into maintaining brain integrity as we age.

**FIGURE 1:**
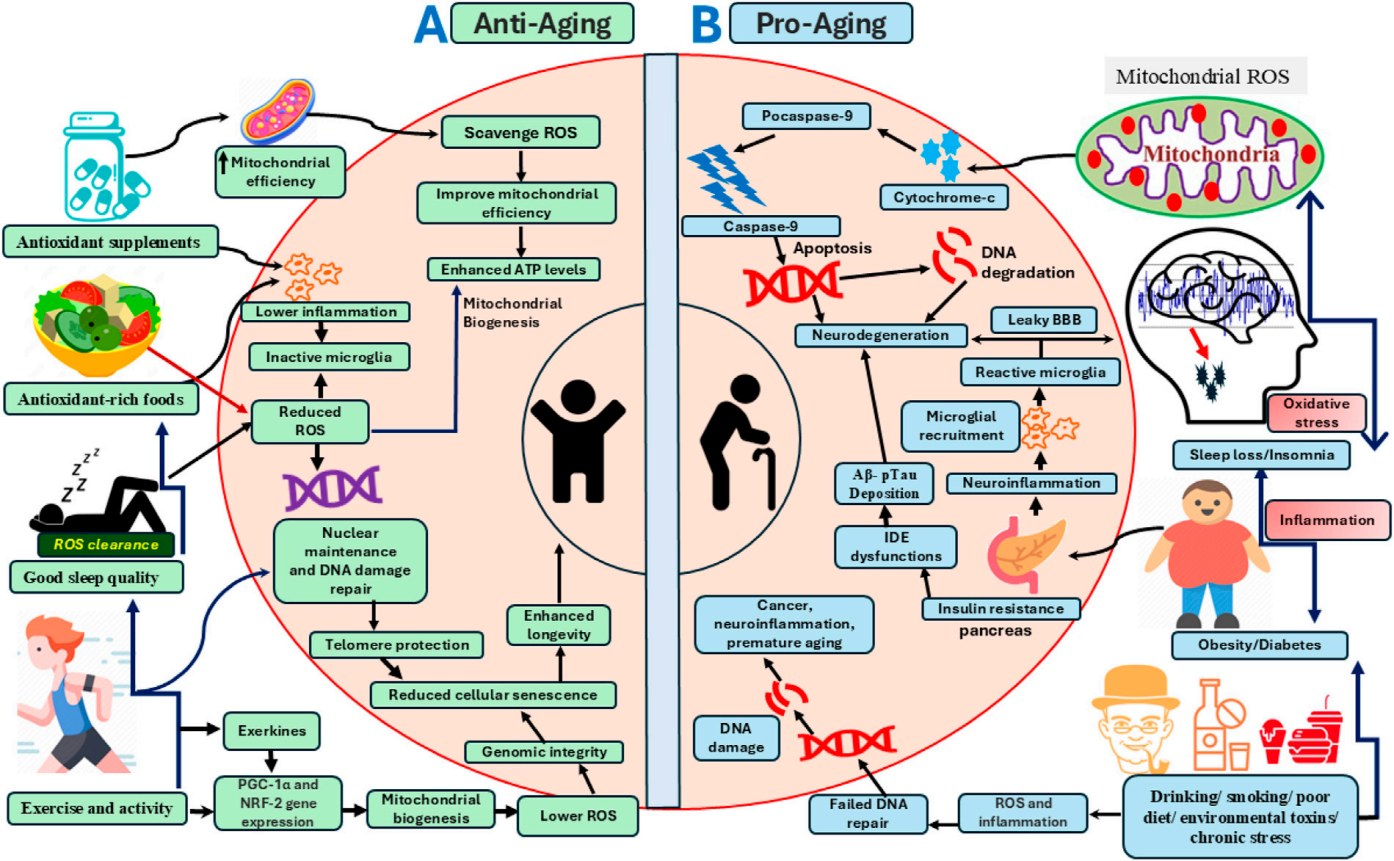
A schematic model of pro-aging and anti-aging pathways modulated by sleep, diet, lifestyle, exercise, oxidative stress, and other metabolic processes at the system and cellular levels. **(A)** The left panel illustrates the effects of interventions and biological processes such as optimal sleep, antioxidant-rich foods, supplements, and exercise in promoting mitochondrial and cellular bioenergetic optimization that enhances longevity. These factors improve free radical scavenging within the cytoplasm and mitochondria, thereby preventing oxidative stress-mediated DNA damage and cellular senescence. Antioxidant ROS scavengers also enhance mitochondrial efficiency by inducing the expression of genes involved in mitochondrial biogenesis, includingPGC-1α (Peroxisome Proliferator-Activated Receptor Gamma Coactivator 1-alpha) and NRF-2 (Nuclear Factor Erythroid 2-Related Factor 2), which together protect against ROS-induced macromolecular damage. Sleep and exercise engage overlapping signaling pathways that regulate mitochondrial biogenesis, redox-sensitive gene expression, and neuroinflammation. Anti-aging factors shown on the left panel also support nuclear maintenance and chromatin remodeling, including DNA repair and preservation of telomere length, thereby promoting cellular health and longevity. **(B)** The right panel depicts pathological aging, which accelerates pro-aging cellular pathways including mitochondrial ROS production, DNA damage, neuroinflammation, and neurodegeneration, ultimately impairing brain health and shortening lifespan. Pathological aging is exacerbated by various factors, including sleep disruption, metabolic disorders (e.g., obesity and diabetes), and adverse lifestyle behaviors such as alcohol consumption, smoking, and diets high in processed foods. Chronic sleep loss, metabolic dysfunction, environmental toxins, stress, and physical inactivity elevate systemic inflammation and ROS-mediated cellular damage. These factors impair mitochondrial function and trigger cytochrome-c release, initiating apoptosis and neurodegeneration. Sleep disturbances and obesity also exacerbate insulin resistance, which promotes neuroinflammation, impairs Aβ clearance, and disrupts blood brain barrier integrity, culminating in neurodegeneration.

In this review, we explore the complex interplay between sleep, metabolism, and aging across five core themes: (1) the role of sleep in maintaining optimal free radical levels, (2) whether ROS directly contribute to or mediate sleep disturbances in aging, (3) how age-related sleep disturbances may, in turn, contribute to impaired brain metabolism and exacerbate age-related changes, (4) the role of sleep, redox, and aging in peripheral systems, and (5) the evidence that improving mitochondrial redox potential restores sleep and slows aging, as well as the potential benefits of interventions like exercise, antioxidant-rich foods, supplements, and specialized diets that enhance sleep and/or metabolic efficiency, and their effects on aging markers (summarized in Figure 1).

## 2 The role of sleep in regulating free radicals in the brain: from Reimund’s free radical flux theory to modern insights

Reimund’s free radical flux theory of sleep, proposed in the 1990s, received significant attention for suggesting that ROS accumulate in the brain during wakefulness, when energy consumption is high, and are subsequently cleared during sleep through a scavenging process that also suppresses their production (Reimund, 1994; Liochev, 2013). This theory suggests that sleep is one of the many antioxidant defense systems employed in mammalian species that restores the redox status of cells and tissues to its equilibrium. Reimund also proposed that oxidative stress acts as a sleep-inducing factor like the catabolic reaction byproduct adenosine diphosphate. In 2007, Savage and West extended this work using mathematical modeling to demonstrate that sleep duration correlates more strongly with brain metabolic rate than whole-body metabolic rate, reinforcing the hypothesis that sleep need is primarily driven by the brain’s metabolic and restorative demands (Savage and West, 2007). Further support of this hypothesis comes from work in *drosophila* suggesting that overexpressing antioxidant genes specifically in the neurons of wildtype flies reduces the amount of time sleeping (Hill et al., 2018). Sleep has many restorative functions and plays a crucial role in scavenging free radicals within the brain. Sleep is categorized into two broad types: non-rapid eye movement (NREM) and rapid eye movement (Mapamba et al., 2022) sleep (Schulz, 2008; Rosenberg and Hout, 2013) based on the power in different frequency bands in the electroencephalogram and level of muscle tone. During NREM sleep the metabolic rate is reduced by approximately 5%–15% compared to wakefulness in the body and brain glucose metabolism also decreases. This creates a restorative period where energy demands are lower, allowing for clearance of metabolic waste like ROS and replenishment of energy stores (Ramanathan et al., 2002; Sharma and Kavuru, 2010; Schmidt, 2014; Aalling et al., 2018; Mir et al., 2019). The hypometabolic state of the brain during NREM sleep inhibits neurons allowing for clearance of toxins, including harmful metabolites and accumulated free radicals during prolonged wakefulness (Xie et al., 2013; Vaccaro et al., 2020). Sleep promotes antioxidant production including melatonin as well as the antioxidant enzymes superoxide dismutase, catalase, and glutathione peroxidase (Gulec et al., 2012; Chang et al., 2016; Monteiro et al., 2024). Finally, repair and regenerative processes during sleep promote efficiency of cellular processes and mitochondrial function, reducing ROS production (Richardson and Mailloux, 2023). Specifically, slow wave activity and delta power during NREM sleep have been shown to promote mitochondrial health, including enhancing mitophagy, increased cisternae surface area (crucial for efficient adenosine triphosphate (ATP) production), and division of mitochondria (Mauri et al., 2022; Hartmann and Kempf, 2023).

Sleep deprivation studies have provided firsthand evidence in support of the free radical flux theory that sleep promotes clearance of overloaded free radicals, protecting the brain against cellular damage and neurodegenerative diseases as well as age-associated pathology (Hill et al., 2018; Trist et al., 2019). Therefore, current therapeutic strategies targeting sleep disturbances in aged individuals could foster resilience against oxidative stress and support overall cognitive function (Davinelli et al., 2024). The bidirectionality of oxidative stress and sleep has also been highlighted in sleep-wake and sleep-breathing disorders where oxidative stress is increased with the progression of pathophysiology. Reports suggest that even one night of sleep deprivation in human subjects alters systemic redox metabolites including plasma antioxidant levels such as glutathione (Trivedi et al., 2017; Chen et al., 2022). Sleep deprivation might also impair the balance of free radical generation by altering mitochondrial metabolic pathways resulting in spiked ROS generation (Cirelli, 2006; Hill et al., 2018; Hartmann and Kempf, 2023).

## 3 Changes in sleep-wake architecture with aging: do reactive oxygen species mediate sleep disturbances in the elderly?

### 3.1 Changes in sleep with aging

Sleep serves a restorative role, allowing the brain and body to recover from the oxidative and metabolic demands of wakefulness. In the elderly, sleep architecture becomes increasingly fragmented, characterized by difficulties in sleep initiation, frequent nocturnal awakenings, and reduced sleep efficiency (Ohayon et al., 2004; Mander et al., 2017). These disruptions are not limited to sleep quantity but extend to qualitative aspects, such as diminished delta power during slow-wave activity, fewer and shorter NREM/REM episodes, and increased latency to these stages (Mander et al., 2017). Importantly, these changes are strongly associated with cognitive decline and age-related neurological disorders (Ohayon et al., 2004; Lupi et al., 2024). In elderly individuals, circadian rhythms as well as sleep-wake regulation are weakened, leading to phase advances, decreased ability to adjust to phase shifts, and reduced amplitude of circadian rhythms (Duffy et al., 2015). These changes are attributed to reduced output of clock gene expression in the suprachiasmatic nucleus (SCN) (Musiek and Holtzman, 2016), neuronal loss, and imbalances in neurohormonal and neurotransmitter systems arising from age-mediated changes in the central nervous system (Zhong et al., 2019). Circadian phase delays and shifting chronotypes to an earlier phase with aging have been associated with shorter telomere length of chromosomes in leukocytes, indicating cellular senescence (Wynchank et al., 2019). Further supporting this notion of the bidirectional relationship between age and sleep-wake disruption, studies have noted that behavioral or genetic manipulation of circadian rhythms also causes aging-like phenotypes (Hood and Amir, 2017). Importantly, age-related changes in sleep-wake patterns exhibit significant sex differences, which are shaped by biological factors (e.g., steroid hormones and genetic differences) as well as environmental and dietary factors (Kostin et al., 2020; Taporoski et al., 2024). In humans, significant sex differences in sleep architecture emerge after middle age, with women more frequently self-reporting sleep disturbances than men. However, the validity of these findings is limited by inconsistencies in objective measurement criteria (van den Berg et al., 2009; Bailey and Silver, 2014; Taporoski et al., 2024). Understanding these sex differences is critical for developing sex-specific geriatric and chronomedicine-based interventions for sleep and age-related disorders (Wiranto et al., 2024). Furthermore, women are approximately 41% more likely than men to develop insomnia or related sleep disturbances, such as delayed sleep onset, difficulty maintaining sleep, and excessive daytime sleepiness, which tend to worsen with age (Zhang and Wing, 2006; Sidani et al., 2019; Alosta et al., 2024). Collectively, these findings confirm that sex significantly influences age-related changes in sleep architecture, driven by sex-specific hormonal, genetic, and physiological factors that must be considered when developing therapeutic strategies for sleep and age-related health conditions.

### 3.2 Age-associated changes in oxidative stress and metabolism

Aging is accompanied by increased oxidative stress due to elevated ROS levels and a concurrent decline in endogenous antioxidant defenses. This redox imbalance disrupts neurochemical signaling and damages sleep-regulating brain regions thereby impairing circadian rhythms and sleep-wake regulation (Carroll and Prather, 2021). Melatonin, a sleep-promoting hormone with antioxidant properties, declines with age, leading to increased mitochondrial ROS production and diminished regulation of the electron transport chain (Wurtman, 2000; Karasek, 2004; Petrosillo et al., 2008; Paradies et al., 2010). Age associated failure of antioxidant machinery such as reduced expression of antioxidant enzymes including catalase, superoxide dismutase and glutathione peroxidase significantly contributes to the age-associated ROS accumulation (Semsei et al., 1991; Finkel and Holbrook, 2000; Barouki, 2006; Gemma et al., 2007). Moreover, sleep deprivation and aging share similar oxidative profiles; both increase ROS accumulation in key regions like the gut and brain, contributing to cellular senescence, neuroinflammation, and cognitive dysfunction (Wang et al., 2010; Vaccaro et al., 2020). This creates a feedback loop in which sleep impairments not only accelerate the aging process by promoting cellular senescence and DNA damage, but also elevate ROS levels, leading to the accumulation of unfolded or misfolded proteins in the endoplasmic reticulum and increased activity of ROS generating enzymes such as NADPH oxidase, xanthine oxidase and phospholipase A2 (Xu et al., 2020). These changes further disrupt antioxidant defense mechanisms in the brain, exacerbating age-related pathologies, including neurodegeneration and memory impairments (Singh and Kumar, 2008; Alzoubi et al., 2012; Villafuerte et al., 2015; Kanazawa et al., 2016; Hill et al., 2018).

Aging profoundly impairs brain energy metabolism and diminishes antioxidant defense mechanisms, leading to excessive accumulation of ROS, which play a central role in sleep disturbances among older adults (Camandola and Mattson, 2017; Błaszczyk, 2020). One key contributor to this metabolic inefficiency is the age-related decline in nicotinamide adenine dinucleotide (Lananna et al.) levels in the mitochondrial salvage pathway, which disrupts the Krebs cycle, reduces ATP production, and impairs DNA repair—cascading into neuronal stress and degeneration (Błaszczyk, 2020). The brain, being highly dependent on glucose and oxygen delivered via cerebral blood flow, becomes especially vulnerable when cerebral blood flow declines due to aging or sleep deprivation, resulting in metabolic instability and overproduction of ROS (Leithner and Royl, 2014; Tarumi and Zhang, 2018; Mokhber et al., 2021; Graff et al., 2023). Accumulated ROS contributes to both sleep deterioration and aging by driving macromolecular damage, including DNA and protein degradation. In addition, elevated ROS levels impair neurogenesis and promote neuroinflammation, which in turn lead to age-associated neurological disorders and cognitive decline (Wang et al., 2010; Yuan et al., 2015; Hill et al., 2018; Chiricosta et al., 2019).

### 3.3 Age-associated changes in circadian rhythms

Age-associated circadian desynchrony has been shown to negatively impact metabolic function, contributing to increased oxidative stress and impaired sleep. However, interventions such as time-restricted feeding can help realign circadian rhythms, reduce oxidative stress, improve sleep quality, and potentially extend lifespan (Tevy et al., 2013; Hood and Amir, 2017; Acosta-Rodríguez et al., 2021). Interestingly, nutrient-sensing pathways which exhibit dynamic responses to dietary interventions, play a central role in aging-related cellular signaling and are also modulated by both circadian rhythms and the sleep-wake cycle (López-Otín et al., 2016; Acosta-Rodríguez et al., 2021). These pathways include pro-aging regulators such as mTOR, PI3K, IGF-1, and AKT as well as anti-aging factors like SIRT1, PGC-1α, and AMPK. Notably, SIRT1 acts as a key integrator of metabolic and circadian signals by regulating core clock genes, including BMAL1 and CLOCK, within the SCN (Tevy et al., 2013). With age, SIRT1 levels decline, disrupting the molecular clock in the SCN and thereby impairing both circadian and metabolic homeostasis (Chang and Guarente, 2013; Acosta-Rodríguez et al., 2021).

### 3.4 Effects of aging and sleep on the glymphatic system and neuron-glial redox coupling in brain health

Aging also impairs the glymphatic system, a crucial brain-wide clearance network responsible for removing interstitial waste products, including neurotoxic and ROS-generating protein aggregates such as amyloid-β, α-synuclein, and neurofibrillary tangles (Rasmussen et al., 2018; Benveniste et al., 2019; Hablitz and Nedergaard, 2021). The glymphatic system primarily functions during NREM sleep, when cerebrospinal fluid influx increases and facilitates the convective removal of metabolic byproducts. This clearance mechanism is essential for maintaining redox balance, as the accumulation of protein aggregates can enhance mitochondrial dysfunction and promote excessive generation of ROS, creating a pro-oxidative and inflammatory environment. With aging, multiple structural and functional alterations such as decreased cerebrovascular pulsatility, reduced aquaporin-4 polarization on astrocyte end feet, and impaired perivascular cerebrospinal fluid exchange significantly reduce glymphatic efficiency. Additionally, age-associated declines in cerebral blood flow and increased blood–brain barrier permeability further compromise glymphatic transport, allowing inflammatory mediators and ROS to accumulate in the brain parenchyma (Banks et al., 2021; Xiong et al., 2024). These alterations not only exacerbate oxidative stress but also fuel chronic neuroinflammation, accelerating neuronal apoptosis and degeneration. Importantly, sleep fragmentation and deprivation themselves have been shown to acutely disrupt glymphatic clearance. Sleep loss decreases the depth and continuity of slow-wave sleep, limiting the time during which glymphatic transport is most active. This creates a vicious cycle, where impaired waste clearance contributes to ROS buildup, which in turn promotes neuroinflammation, alters sleep architecture, and leads to further fragmentation of sleep (Gu et al., 2022; Yi et al., 2022). Studies show that even short-term sleep disruption can reduce the removal of amyloid-β, while chronic sleep loss leads to its accumulation, both of which are strongly associated with increased ROS levels and subsequent damage to neurons.

Sleep disruption not only accelerates the production of ROS but also impairs their clearance by compromising glymphatic system function (Gu et al., 2022). A dysfunctional glymphatic system leads to ROS accumulation across cellular compartments, triggering injury signaling pathways and activating the NLRP3 inflammasome in microglial cells, thereby amplifying neuroinflammation and promoting neurodegeneration and brain aging. Amyloid-β (Aβ) plaques further exacerbate this process by generating ROS after entering mitochondria through the translocase of the outer membrane, causing mitochondrial dysfunction (Chen and Yan, 2007). Sleep deprivation worsens this cascade by hindering glymphatic clearance of Aβ peptides, compounding oxidative damage (Kang et al., 2009; Shokri-Kojori et al., 2018). Glymphatic activity, particularly in clearing metabolic waste, is heightened during sleep. Proteins such as aquaporin-4 are essential for this clearance; aquaporin-4 knockout models demonstrate over a 50% reduction in Aβ removal (Iliff et al., 2012; Kopeć et al., 2023). *Postmortem* analyses of Alzheimer’s disease brain tissue reveal abnormal aquaporin-4 expression and mislocalization, supporting a bidirectional relationship between impaired glymphatic clearance and Aβ accumulation that accelerates brain aging and cellular degeneration (Zeppenfeld et al., 2017; Rasmussen et al., 2018).

Beyond waste clearance, neurons and glia constantly exchange metabolic signals, with glial cells, particularly astrocytes, playing a critical role in redox homeostasis. Astrocytes supply neurons with antioxidants like glutathione and facilitate cerebrospinal and interstitial fluid movement to remove metabolic waste. This astrocyte–neuron redox coupling is highly sensitive to sleep disruption, which alters the brain’s oxidative state and mitochondrial efficiency. Astrocytes exert robust antioxidant effects via the glutathione system and the glutamine, glutamate shuttle, both essential for maintaining neuronal mitochondrial function and ATP production. Disruption of redox coupling can be seen in experimental models where astrocyte-specific mutations in the superoxide dismutase gene lead to enhanced motor neuron degeneration, an effect mitigated by mitochondrial-targeted antioxidants like MitoQ (Cassina et al., 2008). These findings underscore that redox imbalance impairs astrocyte–neuron metabolic interactions, contributing to neurodegeneration and age-related cognitive decline. Finally, emerging evidence suggests a fundamental metabolic divergence between neurons and glia: neurons exhibit lower glycolytic rates and rely more heavily on oxidative phosphorylation than glial cells. This metabolic specialization highlights the importance of glia in supporting neuronal bioenergetics, particularly under conditions of oxidative stress and sleep disruption. In summary, age-related changes in sleep architecture are closely intertwined with elevated oxidative stress and declining antioxidant capacity in the aging brain. ROS emerge as a central mediator in this process, disrupting circadian regulation, impairing glymphatic clearance, altering metabolism and damaging sleep-regulating neural circuits. As sleep disruption further impairs redox balance and waste clearance, a self-reinforcing loop is established thus linking disrupted sleep, oxidative stress, and aging. Understanding these interdependent mechanisms offers critical insight into therapeutic strategies aimed at preserving sleep quality and mitigating neurodegenerative risk in the elderly.

## 4 Sleep disturbances and impaired brain metabolism are closely interconnected, both contributing significantly to the aging process

Sleep is an active process initiated and supported by distinct neural populations within the brain. The aging process and sleep are both significantly influenced by the dynamics of brain bioenergetics. During sleep, the brain undergoes several restorative processes, including energy preservation, DNA repair, tissue repair, clearance of toxins, and rejuvenation of the immune system, ensuring optimal physiological functions. However, as we age, these processes begin to slow down and deviate from their optimal efficiency. Cellular repair mechanisms rely heavily on energy availability. Neuroimaging studies have shown that aging and Alzheimer’s disease brains exhibit a hypometabolic state, which may compromise these repair processes and contribute to accumulating cellular damage. (Womack et al., 2011; Mertens et al., 2021; Xue et al., 2022). Humans spend roughly one-third of their lives sleeping, highlighting the significant role sleep plays in our overall wellbeing (Aminoff et al., 2011). Mitochondria play a crucial role in neurocognitive function and the survival of neuroglial cells by supplying the energy necessary for essential physiological processes. Mitochondrial pathologies include calcium dyshomeostasis, altered mitophagy as well as impaired redox potential which contribute to neurodegeneration and cognitive decline often seen with aging. Interestingly, disrupted sleep has been found to promote mitochondrial dysfunction, increasing oxidative stress in the brain (Hill et al., 2018). Understanding this complex interaction between sleep physiology and brain metabolism could reveal vital insights about aging and its impact on brain health. Prioritizing restorative sleep may be a crucial strategy for supporting healthier aging pathways in the brain.

Since aging is a multifactorial and highly complex biological process, many factors including genetic mutations, epigenetic alterations and metabolic dysregulation play a significant role in the process. Brain metabolism along with sleep impairment play a crucial role in deteriorating cellular and system homeostasis that contributes to cognitive decline as well as aging trajectories (Gonzales et al., 2022; Mukherjee et al., 2024). Current research has significantly explored the bidirectional relationship between dysregulated brain metabolism and sleep impairments in influencing aging as well as age-associated neurocognitive deficits, and suggests that alterations in any one of them exacerbate the other, leading to accelerated aging and neurodegeneration (Bah et al., 2019). Aging has a significant impact on brain metabolism and *vice versa*, since impaired glucose utilization, lipid metabolism as well as oxidative stress in the mitochondria, are altered as we age and conversely, exacerbated ROS and a decline in bioenergetics in neuroglial cells, aggravates aging and cognition (Bratic and Larsson, 2013; Bartman et al., 2024). Additionally, studies from the last decade have suggested that mitochondrial dysfunction caused by mutations in the mitochondrial DNA (mtDNA) as well as impairments in the respiratory chain are the most important molecular determinants of aging and could be vital therapeutic targets (Bratic and Larsson, 2013; Srivastava, 2017). Therefore, age-related diseases and strategies to mitigate them must account for sleep impairments and the restoration of the brain’s bioenergetic balance in order to develop effective therapeutics that slow the aging process. We will review how sleep impairments and brain metabolism affect each other and contribute to the aging process.

### 4.1 Impaired brain metabolism leads to sleep disturbances

Sleep is a physiologically active and regulated state during which consciousness and sensory processing are temporarily reduced to support essential homeostatic functions such as cellular repair, memory consolidation, and metabolic detoxification. Electrophysiologically, sleep is broadly divided into NREM and REM sleep stages, which differ in neurochemical profiles, neuronal activity patterns, and metabolic demands (Savage and West, 2007; Schmidt, 2014). NREM sleep, particularly its deeper stages, is considered a hypometabolic state characterized by reduced cardiorespiratory output, reduced energic demands, and rhythmic slow oscillations. This phase is critical for DNA damage repair, immune memory and homeostasis, synaptic downscaling, and clearance of debris and metabolic waste including byproducts of oxidative stress from brain tissue (Tononi and Cirelli, 2006; Iliff et al., 2012; Xie et al., 2013; Besedovsky et al., 2019; Nedergaard and Goldman, 2020; Zada et al., 2021).

Aging compromises the brain’s metabolic efficiency, primarily due to mitochondrial dysfunction and cumulative oxidative damage to mtDNA (Bhatti et al., 2017; Bartman et al., 2024). These alterations impair ATP synthesis, which is essential for sustaining synchronized neuronal activity during NREM sleep. ATP deficits reduce the ability of sleep-regulating circuits—such as those in the ventrolateral preoptic nucleus, to maintain deep, consolidated sleep, leading to frequent arousals and fragmented sleep patterns in the elderly (Benington and Heller, 1995; Scharf et al., 2008). Interestingly, the ventrolateral preoptic nucleus and other sleep-promoting regions can detect glucose levels in cerebrospinal fluid, further linking central energy sensing to sleep regulation (Mavanji et al., 2015; Varin et al., 2015). Age-related metabolic impairment also disrupts neuro-glial interactions critical for brain homeostasis. For example, astrocyte-neuron metabolic shuttles, such as the glutamate–glutamine cycle, are affected by mitochondrial inefficiency and oxidative stress, reducing synaptic plasticity and impairing glymphatic clearance during sleep (Vyazovskiy and Harris, 2013; Edison, 2024). These dysfunctions not only impact sleep architecture but also accelerate neurodegenerative processes.

In addition to aging, several metabolic disorders underscore the strong bidirectional relationship between disrupted metabolism and sleep impairment. In phenylketonuria, altered amino acid metabolism leads to deficiencies in neurotransmitters like serotonin, dopamine, and norepinephrine, resulting in delayed sleep onset, prolonged latency, and excessive daytime sleepiness (Bruinenberg et al., 2017). Similarly, chronic metabolic diseases such as diabetes impair glucose transport, increase oxidative stress, and trigger neuroinflammation, factors that are known to impair sleep-wake regulation and exacerbate age-related cognitive decline (Muriach et al., 2014; Al-Sayyar et al., 2023). Lifestyle-related metabolic stressors, including alcohol consumption, smoking, obesity, and poor dietary choices, are additional contributors to metabolic dysfunction in the brain and are commonly associated with sleep disorders such as insomnia, sleep apnea, and circadian misalignment (Romero-Corral et al., 2010; Simou et al., 2018; Liu et al., 2021). These conditions further underscore how disrupted brain metabolism, whether due to aging or disease, is a central factor in the pathophysiology of sleep disturbances.

### 4.2 Sleep disruption contributes to dysregulated brain redox and altered metabolism: a bidirectional relationship dictating longevity and neurodegeneration

Sleep plays an essential role in energy optimization and replenishing carbon sources not only for the brain but at a systematic level. Metabolic demands are high during wake to support different body movements, food seeking, reproduction as well as survival of the organism from predators (Ding et al., 2018; Lesku and Schmidt, 2022). Sleep deprivation studies have provided first-hand information about the metabolic changes including the oxidative stress that occurs in the brain as well as peripheral organs as a result of insufficient or total lack of sleep (Vaccaro et al., 2020; Davinelli et al., 2024). Positron emission tomography studies revealed that lack of sleep also causes inefficient glucose utilization especially in higher-order brain regions such as prefrontal cortex, thalamus, hippocampus and cortex that ultimately lead to impairments in cognitive functions (Wu et al., 1991; Shin et al., 2024). Sleep and metabolic disruption including impairments in sleep quality, delta power, spindle density, and altered mitochondrial bioenergetics are intricately linked to aging and age-related dementia (Xie et al., 2013; Zielinski and Gibbons, 2022; Shin et al., 2024). Among several essential factors accelerating age-associated neuropathology, oxidative stress, impaired mitochondrial function, neuroinflammation, and compromised blood-brain barrier integrity are significantly modulated by sleep disruption often observed in elderly subjects (Brunetti et al., 2021; Melhuish Beaupre et al., 2021; Zielinski and Gibbons, 2022). Studies have suggested that the sleep-wake cycle is profoundly affected with age marked by insomnia, sleep fragmentation, and daytime sleepiness that trigger the neurodegeneration and significantly affects the mental health and longevity in elderly subjects (Casagrande et al., 2022; Parhizkar and Holtzman, 2025). Moreover, aging and sleep impairments are co-variable physiological processes that cause a significant reduction in the metabolic efficiency of the brain, especially impacting mitochondrial integrity and function (Tevy et al., 2013; Srivastava, 2017; Zhong et al., 2019). Several studies have reported that targeting metabolic impairments could be a potential tool to mitigate age-mediated changes in sleep, cognition, and longevity by reducing oxidative stress and inflammatory signaling pathways (Brown-Borg et al., 2012; Brunetti et al., 2021; Melhuish Beaupre et al., 2022; Zegarra-Valdivia et al., 2025). Since sleep regulates the brain’s clearance of metabolic toxins such as tau and Aβ-42 peptides via the glymphatic system, age-related sleep disruption may impair this clearance, thereby exacerbating neuronal pathology through increased ROS production and ultimately triggering neuronal apoptosis (Xie et al., 2013; Chong et al., 2022). Therefore, enhancing sleep quality, optimizing metabolism, and reducing ROS may represent promising pharmaceutical targets for managing age-related cognitive decline. These interventions could help mitigate molecular pathologies such as microglial activation, oxidative stress, and mitochondrial dysfunction, which accelerate aging and neurodegeneration, ultimately affecting longevity. Although the complex relationship between oxidative stress and sleep regulation remains under active investigation, numerous studies suggest that wakefulness increases ROS levels, as mitochondria must continuously produce ATP to sustain arousal. This ROS accumulation may, in turn, promote sleep by modulating key signaling pathways (Hill et al., 2018; Davinelli et al., 2024). Studies have linked ROS-mediated gene expression changes in SCN, especially clock and period genes, that influence sleep-wake architecture and cause sleep disturbances (Rutter et al., 2001; Lananna et al., 2018; Davinelli et al., 2024).

### 4.3 Mitochondrial redox signaling as a bidirectional regulator of sleep and cellular homeostasis

Mitochondria are the primary source of intracellular ROS, generating approximately 90% of cellular ROS as byproducts of oxidative phosphorylation (Balaban et al., 2005). Importantly, ROS are not merely metabolic waste, they serve as signaling molecules that regulate essential cellular processes, including autophagy, immune function, differentiation, and responses to hypoxia (Sena and Chandel, 2012; Dai et al., 2014). Emerging evidence also implicates ROS in the regulation of sleep and circadian rhythms. Low levels of ROS promote sleep, whereas excessive ROS disrupt sleep architecture (Ikeda et al., 2005; Wilking et al., 2012; Fagiani et al., 2022). Sleep disruption, in turn, impairs mitochondrial function, reduces antioxidant defenses, elevates ROS production, and leads to the release of mtDNA, calcium dyshomeostasis, and ATP depletion, changes that trigger inflammation and contribute to fragmented NREM sleep (Hartmann and Kempf, 2023; Hu et al., 2024; Zhang Q. et al., 2024).

Sleep loss further compromises mitochondrial health by reducing activity in complexes I and IV of the electron transport chain and dysregulating mitochondrial dynamics. This includes increased phosphorylation of dynamin-related protein 1 (DRP1) at serine-616, promoting mitochondrial fragmentation, and decreased expression of fusion proteins such as mitofusins (MFN1/2) and optic atrophy 1 (OPA1) (Andreazza et al., 2010; Vaccaro et al., 2020; Dai et al., 2021; Mauri et al., 2022; Sarnataro et al., 2025). Sleep deprivation also inhibits mitophagy by suppressing the PINK1/Parkin pathway, allowing the accumulation of damaged mitochondria that further increase ROS and inflammation, accelerating neurodegeneration (Chakravorty et al., 2019; Covarrubias et al., 2021). These mitochondrial impairments are accompanied by reductions in sirtuins, NAD^+^-dependent deacetylases that govern metabolism, stress responses, and circadian gene expression, and altered NAD^+^/NADH ratios (Covarrubias et al., 2021; Zhuang et al., 2024).

In *Drosophila*, a mechanistic link between mitochondrial ROS and sleep regulation has been demonstrated. Sleep deprivation increases mitochondrial ROS in dorsal fan-shaped body neurons, where the β-subunit of Shaker potassium channels (Hyperkinetic) senses redox status via its NADPH cofactor. Oxidation of NADPH slows A-type K^+^ current inactivation, enhances neuronal excitability, and promotes sleep. Genetic or optogenetic suppression of ROS in these neurons reduces sleep, establishing a bidirectional feedback loop: wakefulness elevates oxidative burden, which triggers sleep, and sleep in turn reduces ROS levels (Kempf et al., 2019). Additional mechanisms linking oxidative stress and sleep include the accumulation of extracellular adenosine, resulting from inhibition of adenosine kinase, that activates adenosine A1 receptors to promote sleep (Park and Gupta, 2013; Correia and Vale, 2024). ROS also affect the activity and gene expression of sleep-regulatory neuronal populations, including orexinergic neurons and the SCN, where redox-sensitive transcriptional feedback loops modulate circadian timing (Wilking et al., 2012; Lananna et al., 2018; Pardillo-Díaz et al., 2022). Notably, REM sleep deprivation has been associated with increased mitochondrial biogenesis in the hippocampus and the emergence of manic-like behaviors in rodent models (Kim et al., 2022).

Collectively, these findings support a bidirectional relationship in which sleep promotes mitochondrial redox homeostasis, while mitochondrial oxidative status shapes sleep quantity, quality, and circadian stability, processes with broad implications for aging and neurodegenerative diseases. While *Drosophila* models provide compelling evidence for a causal, bidirectional relationship between oxidative stress and sleep, further work is needed to determine whether these mechanisms are conserved in mammals.

### 4.4 Sleep disruption including jet lag, shiftwork, and sleep apnea contribute to impaired metabolism

Shift work, where an individual’s schedule overlaps with their typical sleep time, is linked to various health issues and contributes to the development of shift work disorder in approximately 25% of affected individuals. This condition is characterized by chronic or recurrent insomnia and excessive daytime sleepiness. Shift-work is associated with cardiovascular disease, diabetes, obesity, cancer, and mood disorders. Studies have shown that limiting food consumption to daytime hours can reduce circadian misalignment, prevent impaired glucose tolerance and pancreatic beta-cell function, reduce cardiovascular risk factors and impaired mood associated with shift work. At the molecular level, feeding during the inactive phase has been shown to abolish the daily rhythm in skeletal muscle mitochondria respiration (de Goede et al., 2022). Moreover, essential mitochondrial processes such as fission, fusion, mitophagy, and NAD + production are regulated or influenced by circadian timing (de Goede et al., 2018). Collectively, these findings suggest that sleep disruption associated with circadian misalignment may impair mitochondrial function and compromise cellular health, leading to redox imbalance and contributing to accelerated aging phenotypes.

Sleep apnea, particularly obstructive sleep apnea, is characterized by recurrent episodes of intermittent hypoxia during sleep due to upper airway collapse. These hypoxic episodes are followed by rapid reoxygenation, which triggers a surge in the production of ROS through mechanisms such as mitochondrial dysfunction and activation of NADPH oxidase pathways (Stanek et al., 2021). This pattern of hypoxemia, hypercapnia, and reoxygenation mimics an ischemia-reperfusion injury, leading to significant oxidative stress and inflammatory responses that have been strongly linked to accelerated biological aging and increased risk of neurodegenerative disease (Li and Wang, 2021). Moreover, intermittent hypoxia leads to repeated micro-arousals, resulting in sleep fragmentation, with individuals experiencing between 10 and 43 micro-arousals per hour (Martin et al., 1997). These frequent arousals disrupt the continuity and depth of slow-wave sleep, reducing the restorative functions of sleep and further impairing glymphatic clearance of metabolic waste products, including ROS-generating aggregates such as beta-amyloid. Over time, this results in increased ROS accumulation, neuronal stress, and a decline in redox homeostasis. High-frequency sleep fragmentation in obstructive sleep apnea has been shown to induce outcomes similar to chronic sleep deprivation, including reduced antioxidant enzyme activity (e.g., superoxide dismutase, glutathione peroxidase), aberrant mitochondrial morphology, and diminished ATP production, all of which impair neuronal metabolism (Bonnet and Arand, 2003). These metabolic disturbances contribute to neuronal inflammation, cellular senescence, and cognitive dysfunction, hallmark features of brain aging.

## 5 Sleep and mitochondrial health affect peripheral systems including the immune system and gut

### 5.1 Mitochondrial decline, inflammaging, and sleep

The concept of “inflammaging,” first introduced by Claudio Franceschi in 2000, refers to the chronic, low-grade systemic inflammation that characterizes biological aging (Franceschi et al., 2000; Franceschi and Campisi, 2014). This age-associated inflammatory state arises from a complex interplay of internal and external stressors, including poor diet, psychosocial or physical stress, environmental toxins, persistent infections, and disrupted circadian rhythms. Chronic exposure to these factors leads to sustained immune cell activation, which in turn drives mitochondrial dysfunction and elevated ROS levels further amplifying immune activation and inflammatory signaling (Allen et al., 2021; Kaliszewska et al., 2021; Li et al., 2021; Shang et al., 2021; Reddam et al., 2022; Pollicino et al., 2023).

Mitochondria play a pivotal role in immune cell metabolism and function. As immune cells age, particularly T cells, mitochondrial efficiency declines, leading to reduced ATP production, increased ROS generation, and impaired mitophagy. These changes drive T cells toward senescence, a state of irreversible cell cycle arrest accompanied by the senescence-associated secretory phenotype, characterized by inappropriate activation, poorer function and enhanced secretion of proinflammatory cytokines such as IL-6, TNF-α, and IFN-γ (Olivieri et al., 2018; Lee et al., 2022). This not only contributes to systemic inflammation but also impairs immune surveillance and responsiveness. Experimental models have further highlighted the causal role of mitochondrial dysfunction in immune aging. For instance, targeted deletion of mitochondrial transcription factor A, known as TFAM, is important for mtDNA stability leading to decreased mtDNA copy number and impaired oxidative phosphorylation. This results in elevating ROS and oxidative damage ultimately inducing early mitochondrial failure in T cells. This mitochondrial failure results in the immune senescence of those same T cells and a systemic accelerated aging phenotype, marked by motor deficits, cardiovascular dysfunction, and cognitive decline (Desdín-Micó et al., 2020). These findings underscore the centrality of mitochondrial health in maintaining immune competence and delaying age-related degeneration.

Sleep supports mitochondrial restoration through enhanced antioxidant defenses, autophagy, and bioenergetic recovery (Mauri et al., 2022; Richardson and Mailloux, 2023; Lei et al., 2024). In immune cells, these processes are essential for preventing metabolic exhaustion and maintaining immunological vigilance (Angajala et al., 2018; Steinert et al., 2021). Sleep, particularly deep slow-wave NREM sleep, emerges as a critical modulator of immune function and a potential buffer against inflammaging. Beyond its restorative effects, sleep actively shapes immune responses and is essential for immunological memory consolidation. During NREM, antigen-presenting cells interact more effectively with helper T cells to reinforce adaptive immune responses. This process resembles memory consolidation in the brain and is thought to optimize long-term immune defense (Lange et al., 2011; Besedovsky et al., 2012; Besedovsky et al., 2019). Hence, sleep disruption may not only impair the acute immune response but also weaken the long-term immunological repertoire, increasing susceptibility to infections and impairing vaccination efficacy.

The immune system is also a critical regulator of sleep. Cytokines such as IL-1β and TNF-α, which are pivotal in inflammation, also serve as sleep-regulating molecules, promoting NREM sleep at physiological levels but contributing to sleep fragmentation when chronically elevated (Besedovsky et al., 2019; Zielinski and Gibbons, 2022). Conversely, sleep deprivation skews immune balance, reducing the number and function of natural killer cells (De Lorenzo et al., 2015), impairing T-cell responsiveness (Tune et al., 2021), and elevating circulating proinflammatory cytokines, thus mimicking an inflammaging-like profile even in younger individuals (Garbarino et al., 2021).

Sex-based differences in mitochondrial physiology add an important layer of complexity when considering mitochondrial-targeted interventions for sleep, aging, and age-related inflammation. Studies in Fischer rats have shown that female skeletal muscle exhibits greater mitochondrial resilience and sustained function following oxidative stress, likely due to the protective effects of estrogen (Farhat et al., 2017). In sedentary animals, female muscle fibers maintained more efficient respiration and ATP production in response to ROS compared to males; however, this advantage diminished with exercise, where males showed improvements, indicating a sex-specific interplay between physical activity and redox regulation. Estrogen is proposed to exert its effects through activation of the MAP kinase pathway and transcription factor NF-κB, leading to increased expression of antioxidant enzymes such as superoxide dismutase (Farhat et al., 2017; Guajardo-Correa et al., 2022). It also enhances mitochondrial respiratory chain activity, improves membrane potential, and reduces hydrogen peroxide production (Stirone et al., 2005; Klinge, 2020). Moreover, nuclear–mitochondrial genome interactions differ by sex, influencing longevity and stress adaptation, with females exhibiting more robust mitochondrial maintenance—again, largely attributed to estrogenic signaling (Tower, 2015). Together, these findings underscore significant sex differences in mitochondrial function that contribute to disparities in aging trajectories, cellular senescence, sleep impairments, and dementia risk. As such, sex should be carefully considered when developing mitochondrial-targeted therapeutics or supplements for aging and sleep-related disorders.

In summary, the immune system is a pivotal mediator of aging where disruptions in sleep and mitochondrial function form a self-reinforcing loop that accelerates inflammaging processes. Mitochondria are at the nexus of this interaction, both as generators of ROS and as regulators of immune cell function. Sleep, by modulating redox balance and supporting immune homeostasis, acts as a critical counterbalance. Disruption of this equilibrium, whether through poor sleep, chronic inflammation, or mitochondrial dysfunction, propels the aging process and increases vulnerability to age-related diseases. Addressing sleep quality, mitochondrial health, and sex differences may therefore represent a powerful strategy to delay immunosenescence and mitigate inflammaging.

### 5.2 The gut’s role in mediating sleep and redox balance during aging

The gastrointestinal tract has long been studied within the context of aging and exhibits multiple hallmarks of physiological decline, including altered microbial colonization, elevated low-grade inflammation, compromised barrier integrity, and reduced absorptive capacity. These changes contribute to broader issues such as nutritional deficiencies, systemic inflammation, and gut dysbiosis in aging individuals. Importantly, sleep disorders and circadian misalignment have been shown to further disrupt gut microbiota composition, leading to altered microbial metabolite profiles (Lin et al., 2024), impaired antioxidant production, and increased generation of ROS. Together, these fuel inflammatory cascades and metabolic dysfunction. More recently, the gut has emerged as a central organ at the intersection of sleep, redox biology, and aging (Homolak, 2023). Groundbreaking work by Vaccaro et al. (2020) demonstrated that chronic sleep deprivation in *drosophila* and mice induces the most dramatic increase in ROS not in the brain, but in the gut. Intriguingly, lifespan under sleep deprivation conditions could be extended by the gut-specific expression of antioxidant enzymes, whereas similar antioxidant interventions in the brain had no protective effect. These findings suggest that gut redox homeostasis is a key beneficiary of the restorative processes associated with sleep but that the gut may also actively mediate the systemic effects of sleep, including on longevity and aging (Vaccaro et al., 2020).

The gut, being the largest mucosal interface between the external environment and internal physiology, is a unique redox-sensitive organ. Unlike the brain and other high-oxygen tissues where ROS generation is largely linked to mitochondrial oxidative phosphorylation, the gut’s redox balance is shaped by a more complex interplay between host mitochondrial metabolism, microbial ROS/antioxidant production, and immune cell activity. Aging disturbs this delicate balance through mechanisms including impaired epithelial turnover, immune senescence, and shifts in microbial populations that favor pro-oxidant species. These age-related changes impair mucosal integrity and nutrient sensing, fostering a proinflammatory gut environment that can accelerate systemic aging processes. Recent studies have also identified microbial-host co-metabolites that link gut dysbiosis to sleep and aging. For instance, phenylacetylglutamine, a metabolite derived from microbial metabolism of dietary phenylalanine, has been associated with short sleep duration, mitochondrial dysfunction, and age-related comorbidities in humans (Fritz et al., 2023; Krishnamoorthy et al., 2024; Yang et al., 2025). Mechanistically, phenylacetylglutamine has been shown to induce mitochondrial stress and elevate ROS levels, leading to cellular senescence and tissue dysfunction (Yang et al., 2025). This suggests that microbial metabolites can directly influence host redox signaling and aging trajectories, potentially linking sleep disturbance-induced dysbiosis to long-term health consequences. Altogether, these findings underscore the gut as a metabolically and immunologically active organ whose redox homeostasis is central to the aging process, and which is highly sensitive to disruptions in sleep architecture. The feedback loop between poor sleep, gut dysbiosis, redox imbalance, and aging highlights a promising target for future interventions aimed at promoting healthy aging and metabolic resilience.

## 6 How exercise, antioxidant-rich foods, and supplements improve sleep, boost metabolic efficiency, and support longevity

Aging is a gradual loss of optimal physiological processes with accumulated cellular and molecular damage that cannot be reversed but certain factors, both intrinsic and extrinsic, play an important role in its acceleration or slowing it down to delay cellular senescence and aging (Tenchov et al., 2024). The fast-growing socioeconomic uncertainties over the health burden of age-related diseases has put significant research focus on the science of aging and longevity. Gerontologists and ancient philosophers alike have long pondered ways to reverse or slow the aging process, a pursuit that continues into the 21st century, now empowered by cutting-edge technology. In addition to the genetic basis of longevity and metabolic efficiency, lifestyle factors including diet, sleep, stress, exercise as well as supplements are essential tools to mitigate and improve longevity. The world population above 60 years old will cross 2 billion by the end of 2050 making almost one-fourth of the global population “aged and vulnerable” to multiple age-associated comorbidities (Officer et al., 2016; Tenchov et al., 2024). With this there will be a rise in age-related health conditions including but not limited to Alzheimer’s disease, cardiovascular, metabolic, cancer as well as other diseases that typically double in incidence after every 5 years after 60 years old (Melzer et al., 2020). The history of anti-aging research is rich and stretches back to ancient times, with early physicians and philosophers from civilizations like Rome and China developing a variety of strategies. These included herbal remedies, acupuncture, and specialized diets featuring foods such as cabbage and berries, which were believed to slow the aging process. We will now examine briefly how exercise as well as antioxidant-rich diets along with supplements improve brain metabolism, thereby improving sleep and slowing age-associated diseases. The cellular mechanisms behind exercise and diet induced improvements in sleep and longevity are still being determined. Cutting-edge technologies have advanced our understanding regarding the signaling pathways that are activated by physical exercise and specific nutrient uptake rich in antioxidants.

### 6.1 Evidence supporting the role of exercise in regulating ROS, enhancing sleep, and promoting longevity

Regular physical exercise offers multiple health benefits, including improved sleep quality, regulation of blood glucose levels, and mitigation of oxidative stress and metabolic dysfunction in both humans and animal models. These include optimal cardiovascular health and reduced risk of age-related diseases like cancer, neurodegeneration promoting longevity (Simioni et al., 2018). Physical exercise has been shown to enhance the production of melatonin which is an antioxidant and promotes sleep (Kruk et al., 2021; Alnawwar et al., 2023). The indoleamine structure of melatonin helps it exert antioxidant as well as anti-inflammatory effects in addition to acting on the SCN (the circadian master clock) to synchronize day-night rhythms. However, the relationship between physical exercise and melatonin production remains complex and not fully understood. While exercise induces a mild increase in oxidative stress due to heightened energy demands, melatonin counteracts this effect by serving as a potent antioxidant and reducing oxidative stress (Kruk et al., 2021; Alnawwar et al., 2023). The type and duration of exercise, chronic *versus* acute, short term *versus* long term, and timing of exercise are other factors that dictate whether the oxidative stress is elevated or mitigated. However, the general consensus is that long-term and moderate intensity exercise decrease ROS production and also mitigate the age-mediated decrease in antioxidant defense mechanisms and mitochondrial dysfunction (Bouzid et al., 2018; Jia et al., 2023). Physical exercise stimulates tissue rejuvenation and repair as well as resets the circadian rhythms altered by aging and age-mediated diseases especially cardiovascular, neurodegeneration, cancer, obesity and liver dysfunction that are primarily due to impairments in metabolic homeostasis (Silva et al., 2021; Jia et al., 2023). Age-related sarcopenia with a significant loss of skeletal muscle and overall body weight is a significant factor contributing to loss of physical vigor in elderly. Physical exercise has been shown to mitigate sarcopenia and improve vascular health in the elderly largely by influencing inflammatory pathways as well as clock genes that regulate circadian rhythms and sleep-wake physiology (de Sá Souza et al., 2022). Aging significantly elevates ROS and inflammation that together exacerbate cardiometabolic and vascular diseases. Intense exercise including resistance training decreases both ROS and inflammatory markers including interlukin-6, C-reactive protein and tumor necrosis factor-alpha (Sardeli et al., 2018). Exercise optimizes the antioxidant defense pathways and simultaneously prevents protein oxidation and lipid peroxidation both adults as well as the elderly (Bouzid et al., 2018). These studies highlight the fact that exercise acts on various cell signaling levels to limit the generation of ROS as well as enhance its clearance when accumulated.

The literature on exercise as an intervention is extensive and has demonstrated benefits across multiple systems affected by aging including the immune system and gut (Mattson and Arumugam, 2018; Vecchio et al., 2018). One of the broadest benefits of exercise is its ability to improve redox balance by upregulating antioxidant genes, enhancing mitochondrial quality and function (Steiner et al., 2011; Marques-Aleixo et al., 2015), and modulating redox-sensitive signaling pathways. The improvement in redox balance has important downstream effects, such as reducing DNA damage, lowering chronic immune activation, and improving vascular function.

In the brain, exercise mitigates aging-related decline by supporting cellular repair and regenerative processes and enhances molecular waste disposal. Exercise supports neuroplasticity and repair by enhancing brain-derived neurotrophic factor production (Yang et al., 2013; Szuhany et al., 2015), stimulating DNA repair processes (Yang et al., 2013; Soares et al., 2015; Vilela et al., 2020), and boosting the expression of neurogenesis-related and proliferative genes in aged animals (Buckley et al., 2023). Crucial factors in aging are the reduction of autophagy (Andreotti et al., 2020) and glymphatic clearance. Exercise improves glymphatic function and molecular waste disposal by reduced activation of glia, resulting in better clearance of amyloid-β (He et al., 2017) and enhanced aquaporin 4 expression and localization to astrocytic end feet. Interestingly, glymphatic clearance can be enhanced even during wakefulness, as von Holstein-Rathlou et al. (2018) demonstrated increased glymphatic flux during daytime, non-exercise awake periods, suggesting a role for circadian regulation beyond sleep-dependent mechanisms. (von Holstein-Rathlou et al., 2018).

Moderate exercise is associated with improved immune function and reduced cellular senescence. It enhances key immune responses, including natural killer cell cytotoxicity, neutrophil phagocytosis, and monocyte activity (Simpson et al., 2015) while also promoting immunosurveillance (Baskerville et al., 2024). Additionally, moderate exercise contributes to reduced immune senescence by rejuvenating aging immune cells (Lee et al., 2019), decreasing the number of senescent cells (Spielmann et al., 2011), and suppressing signaling pathways that promote stem cell exhaustion (Liu et al., 2023).

The role of exercise in promoting gut health during aging is an emerging but still underdefined area of research. Aging is commonly associated with gut dysbiosis, marked by reduced microbial diversity and a decline in beneficial bacterial populations, which has been linked to cognitive decline and increased frailty (Claesson et al., 2012; O’Toole and Jeffery, 2015; Salazar et al., 2017). While several studies suggest that regular exercise can enhance microbial diversity, increase secretory IgA levels, and promote short-chain fatty acid production, benefiting gut barrier integrity, reducing inflammation, and improving motility, these effects appear less consistent in older adults (Clauss et al., 2021). Interventions in elderly populations have shown more variable and often attenuated outcomes (Ramos et al., 2022; Hernández-Urbán et al., 2023). This variability may stem from differences in baseline microbiome composition, exercise type, intensity, frequency, and individual factors such as age, diet, and overall health status, complicating the interpretation and generalization of findings.

### 6.2 Evidence for antioxidant rich foods/diets in regulating ROS, improving sleep, and promoting longevity

Growing evidence from recent studies has shifted the focus from sleep-promoting drugs with latent side effects to different antioxidant-rich foods and dietary strategies, including fasting, supplements, and ketogenic diets. These may improve sleep as well as restore metabolic dysregulation, thereby mitigating age-associated health conditions. Recent studies suggest that varying the macronutrient fat and carbohydrate composition impacts the quality of sleep, especially NREM sleep, delta power, and the frequency of REM sleep (St-Onge et al., 2016; Mantantzis et al., 2022). Since oxidative stress is prevalent in insomnia patients as well as those with sleep-related disorders (Bin Heyat et al., 2022), antioxidant rich foods and ketogenic diets have been shown to improve sleep patterns. They reset metabolic dysregulation that in turn promotes longevity by minimizing cardiovascular disease (Pietrzak et al., 2022; Sarode and Nikam, 2023). The buildup of free radicals with aging as well as sleep loss, exacerbates the metabolic dysfunctions and risks for age-related morbidity due to cardiovascular and neurodegenerative diseases (de Almeida et al., 2022; David et al., 2023). Foods that are rich in antioxidants such as vitamins, polyphenols, flavonoids, carotene and minerals have been shown to mitigate age-associated pathologies in human and animal models by reducing the cellular damage mediated by ROS (Bakoyiannis et al., 2019; Rusu et al., 2020; Wang et al., 2023). This signifies the need for maintaining good and balanced dietary practices that largely include consuming whole nutrient dense foods including fruits, nuts, seeds, vegetables, green tea and spices like turmeric that have abundant amounts of antioxidants (Jiang and Xiong, 2016; David et al., 2023) which have been shown to decrease age-related illnesses (Jiang and Xiong, 2016; St-Onge et al., 2016; Katsube et al., 2022; David et al., 2023). These studies suggest that foods rich in antioxidants protect against age-related diseases, improve metabolism and enhance sleep quality that together act to increase longevity.

Studies using the ketogenic diet, which is high in fat and low in carbohydrate, have shown that effective implementation of the ketogenic diet regimen offers significant neuroprotective benefits, including improved cognition in different psychiatric disorders and decreased age-related symptoms (Hallböök et al., 2012; Choi et al., 2024). Interestingly, various cellular signaling pathways modulated by the ketogenic diet are implicated in sleep-wake regulation as well as in circadian rhythm dynamics. The ketogenic diet improves NREM sleep and resets the programming of circadian rhythms mediated by the ketones bodies acetoacetate and β-hydroxybutyrate (O’Hearn, 2021; Merlino et al., 2023). The ketogenic diet has a direct effect on metabolism as the ketones generate fewer free radicals than glucose in the mitochondria. Thereby preventing ROS-mediated damage to cells and providing efficient ATP supply to neurons, both necessary to support optimal sleep quality (Maalouf et al., 2007; Robberechts et al., 2023). Moreover, the ketogenic diet has been shown to improve and significantly increase median lifespan as well as survival in mice compared to the control diet by enhancing and preserving motor function and cognition as well as preventing sarcopenia and tumor development in aged mice (Roberts et al., 2017). Sleep deprivation and aging are known to upregulate histone deacetylases, which remove acetyl groups from histone proteins, leading to chromatin condensation and suppressed expression of genes critical for long-term potentiation and synaptic plasticity (Wong et al., 2020). The ketone beta-hydroxybutyrate extends lifespan and improves cognition in yeast and fly models by directly inhibiting histone deacetylases as well as reducing ROS (Shimazu et al., 2013; Newman and Verdin, 2014). Since inflammation, alongside oxidative stress, is a major contributor to aging and age-related neurodegeneration, ketone bodies have also been shown to reduce both oxidative stress and inflammatory markers, thereby promoting longevity and alleviating age-associated health issues (Pinto et al., 2018; Monda et al., 2024). Collectively, these studies highlight the positive health effects of ketogenic diets, particularly in enhancing longevity and cognitive function in the elderly.

In addition to antioxidant-rich foods and ketogenic diets, another dietary intervention that has shown many benefits in reducing age-related disease burden and improving metabolism is fasting. Fasting resets neuroimmunomodulation, enhances autophagy and clearance of dead tissues, and restores metabolism, thereby helping to increase lifespan (Shabkhizan et al., 2023). Various fasting regimens are currently being studied to improve health, including alternate-day fasting, intermittent fasting, 5:2 where one eats normal healthy meals 5 days and a very calorie restricted diet 2 non-consecutive days a week, 16:8 time restricted eating where one fasts for 16 h a day and only eats during an 8 h window. These fasting interventions improve metabolic health (Różański et al., 2021). Alternate-day fasting in middle-aged humans improves physiology and reduces markers of aging and metabolic impairments (Stekovic et al., 2019). Metabolic dysregulation such as insulin resistance and diabetes are often associated with obesity and are comorbid factors for cardiovascular disease, inflammaging, and shorter lifespan. Fasting has been shown to improve these factors, thereby delaying aging and age-related disorders (Longo and Mattson, 2014; Sutton et al., 2018; Hardiany et al., 2022). Interestingly, a study found that a short-term modified fasting regimen in middle-aged individuals reduced sleep arousals and periodic leg movements. It also showed a trend to increased REM sleep, suggesting potential benefits to overall sleep quality (Michalsen et al., 2003). Overall, there is no clear consensus on the effects of fasting on sleep and wakefulness. While some studies report improved sleep quality, others have found reductions in sleep duration (Bohlman et al., 2024; Kerkeni et al., 2024). These discrepancies may be due to variations in fasting regimens and individual factors such as metabolic health, age, sex, and race. Future research is needed to clarify the underlying mechanisms and cell signaling pathways influenced by different types of fasting protocols.

### 6.3 Evidence supporting the role of antioxidant supplements in regulating ROS, enhancing sleep, and promoting longevity

More than half of United States adults buy supplements containing antioxidants including vitamins A, C, and E, as well as lycopene, glutathione, flavonoids, lutein, and resveratrol aiming to improve health (Poljsak et al., 2013). The effectiveness of antioxidants in mitigating age-associated pathology and improving sleep is still debated and an active area of research. An ideal antioxidant must meet certain criteria to be effective including a fast absorption rate, chelating redox metallic compounds, work in cell membranes as well as in aqueous medium, and induce changes in gene expression to help cells mitigate ROS (Rahman, 2007; Di Meo et al., 2016). Antioxidants can be synthetic or natural biomolecules that neutralize free radicals and offer cellular protection against ROS-mediated senescence. The use of antioxidant supplements to slow aging and promote optimal health is gaining traction, driven by their unique properties and anti-inflammatory benefits. However, studies investigating their effectiveness in aging remain inconclusive, as ROS also play essential physiological roles, including functioning as signaling molecules. Moreover, excessive use of certain antioxidants may disrupt these processes and potentially cause harm to cellular function (Poljsak et al., 2013; Di Meo et al., 2016). Several antioxidants have improved sleep quality and reduced oxidative stress. For example, the mushroom-derived antioxidant ergothioneine mitigates anxiety and improves sleep quality in human subjects in a 4-week trial period (Katsube et al., 2022). Antioxidant supplements hold significant potential in mitigating ROS-mediated cellular and tissue damage, either by directly neutralizing free radicals, as seen with vitamins C and E, or by supporting enzymatic defense systems such as superoxide dismutase and catalase, which actively scavenge free radicals (Poljsak et al., 2013; Liu et al., 2018). Antioxidant supplements contain various macronutrients, such as amino acids, and micronutrients that individually or synergistically target ROS molecules, aiding in their neutralization and helping restore a balanced redox state that minimizes cellular risk.

Several studies have shown that antioxidant supplements can alleviate sleep problems in both young and elderly populations, primarily by targeting ROS-generating pathways and scavenging free radicals, thereby preventing cellular damage (David et al., 2023; Jiang et al., 2024). Many of these supplements are derived from naturally occurring polyphenolic compounds, such as flavonoids, which have been reported to reduce oxidative stress and neuroinflammation (Bakoyiannis et al., 2019). Current evidence suggests that polyphenol-based dietary supplements containing flavonoids, including astragalin, may enhance sleep duration, decrease sleep latency, and improve overall sleep quality, ultimately reducing the health burden associated with sleep disorders (Li et al., 2017; Wang et al., 2023). Additionally, a randomized controlled trial found that isoflavone-based supplements improved sleep quality and reduced insomnia symptoms in postmenopausal women, further supporting a strong link between antioxidant supplementation and improved sleep outcomes (Hachul et al., 2011). Another widely used antioxidant supplement for sleep enhancement is melatonin, a naturally occurring hormone that regulates the sleep-wake cycle and also functions as a potent antioxidant. Although exogenous melatonin has not been officially approved by the United States Food and Drug Administration, it is available over the counter as a synthetic dietary supplement that mimics the effects of endogenous melatonin. However, it is important to note that excessive use of antioxidant supplements can lead to “antioxidant stress,” which may counteract their benefits and potentially contribute to metabolic disorders, including cancer (Mondul et al., 2011; Jabbari et al., 2024). A randomized chemoprevention trial in Finland involving of 29,133 male smokers aged 50–69 years found that beta-carotene supplementation increased the risk of lung cancer compared to placebo, highlighting how antioxidant effects can be influenced by confounding lifestyle factors such as tobacco and alcohol use (Middha et al., 2019). Similar concerns apply to other supplements, including melatonin, where long-term safety remains an area of active investigation. Thus, while antioxidant supplements may offer benefits for sleep and oxidative stress, their use should be guided by evidence-based recommendations to minimize potential adverse effects.

## 7 Current challenges, future perspectives, and conclusions

This review explores the complex relationship between sleep, redox metabolism, and aging, highlighting their critical role in brain health and longevity (Figure 1). Sleep is essential for regulating metabolism, clearing free radicals, and maintaining neuronal function. However, aging disrupts mitochondrial redox balance, leading to increased oxidative stress, sleep disturbances, and a heightened risk of neurodegenerative diseases like Alzheimer’s disease. Studies suggest that interventions such as antioxidant-rich diets, sleep-enhancing drugs, and exercise can improve mitochondrial function, mitigate oxidative damage, and promote healthier aging. By understanding these interactions, researchers can develop targeted therapies to reduce age-related diseases and improve overall wellbeing.

Oxidative stress and mitochondrial dysfunction are central features of aging, sleep-wake disruptions, and neurodegenerative disorders such as Alzheimer’s disease. As mitochondrial efficiency declines and ROS accumulate with age and disease progression, cells experience increased molecular damage and impaired neurophysiological function, including disrupted sleep architecture. Antioxidant interventions, whether through supplementation or diets rich in polyphenols, flavonoids, and carotenoids, hold promise for restoring mitochondrial function, reducing oxidative damage, and improving sleep and cognitive outcomes. While experimental evidence in animal models and some human studies supports the potential benefits of antioxidant strategies for mitigating age-related pathologies, translation to clinical practice remains challenging. Variability in study outcomes is influenced by dose, timing, source (synthetic vs. natural), individual health status, and biological variables such as sex and genetics. These complexities underscore the need for caution and precision in the clinical application of antioxidant supplements.

Future research should prioritize integrative, systems-level approaches to unravel how antioxidants influence mitochondrial bioenergetics, redox signaling, and sleep regulation across diverse populations. Longitudinal cohort studies and well-controlled clinical trials are needed to examine sex- and race-specific responses, as well as the impact of genetic variation in antioxidant pathways. New technologies, such as single-cell multiomics, mitochondrial transcriptomics, and real-time *in vivo* imaging of ROS and calcium, offer powerful tools to dissect the spatial and temporal dynamics of antioxidant effects. Importantly, antioxidant therapies may be most effective when combined with lifestyle interventions. Approaches such as time-restricted feeding, exercise, natural antioxidant-rich diets, and sleep hygiene practices may act synergistically to support mitochondrial health and promote healthy aging. Interdisciplinary research aimed at decoding the mechanistic links between antioxidants, metabolic and inflammatory signaling, and aging biology will be essential to develop personalized, evidence-based strategies for enhancing sleep, cognition, and longevity.

Studying redox biology in the context of sleep and aging presents several challenges due to the complexity of these interconnected processes. One of the primary difficulties lies in the dynamic and context-dependent nature of redox signaling, making it hard to establish clear causal relationships between oxidative stress, sleep regulation, and aging. Additionally, measuring real-time redox changes poses a significant challenge for future studies, as ROS and antioxidants operate on very short timescales and in localized cellular compartments, requiring highly specialized techniques for accurate assessment. Interindividual variability further complicates research, as aging and sleep patterns differ widely among individuals, making it difficult to generalize findings and establish universal mechanisms. Furthermore, while animal models provide valuable insights, differences in sleep architecture and metabolic processes between species can limit the direct applicability of these findings to humans. The bidirectional relationship between sleep and redox biology also presents a challenge, as sleep influences redox balance, while oxidative stress, in turn, affects sleep patterns, making it difficult to determine cause and effect.

Lifestyle factors such as diet, exercise, and environmental exposures further complicate the study of redox regulation in sleep and aging, as they significantly influence both oxidative metabolism and sleep architecture. These confounding variables make it difficult to isolate intrinsic effects of aging in experimental models. Technological limitations also persist, as non-invasive methods for assessing oxidative stress in the human brain during sleep are still under development, restricting real-time, *in vivo* observations. Although fluorescent ROS-sensitive dyes (e.g., dihydroethidium) and genetically encoded probes (e.g., Grx1-roCherry) allow *postmortem* ROS detection in animal models (Hosoi et al., 2019; Shokhina et al., 2019), translating these tools for human use remains a major challenge. Advancements in ROS imaging, such as bioluminescent sensors, protein-based reporters, and hybrid modalities like PET-ROS imaging, are emerging but remain limited by sensitivity, specificity, and tissue accessibility (Boutagy et al., 2018). Human studies currently rely on indirect markers of oxidative stress in plasma, urine, or tissue biopsies, such as 8-hydroxy-2′-deoxyguanosine (8-OHdG), which primarily reflect DNA oxidation while overlooking protein and lipid oxidative damage (Hawkins and Davies, 2019; Larsen et al., 2019; Murphy et al., 2022). A recent proof-of-concept study demonstrated *in vivo* ROS imaging by applying cold atmospheric plasma to the skin of mice injected with TEMPOL, then detecting hydroxyl radical oxidation products using dynamic nuclear polarization–MRI and electron paramagnetic resonance; however, such techniques remain technically demanding and restricted to superficial tissues (Badr et al., 2024). Importantly, redox biology does not operate in isolation, it intersects with key aging-related pathways including inflammation, autophagy, and energy metabolism. This mechanistic overlap complicates efforts to parse the independent contribution of oxidative stress to sleep and aging. Moving forward, progress in this field will require integrative, multi-disciplinary approaches that leverage advanced imaging techniques, longitudinal studies, and systems-level tools such as genetics, metabolomics, and sleep phenotyping. These efforts will be critical to unravel the complex interplay between sleep, redox biology, and aging, and to develop targeted interventions that promote healthy longevity.
